# Supplementary material for: Topological Distribution of the Sex Hormone Receptor Expressions Highlights the Importance of Stromal ERα and Epithelial PR in Malignant Transformation of the Uterine Cervix
Source: Int J Mol Sci. 2025 May 6;26(9):4418. doi: 10.3390/ijms26094418 (PMC12073007; doi:10.3390/ijms26094418)
Supplement: Supplementary file 1 [file ijms-26-04418-s001.zip › Table S1.pdf]

Table S1. Status of ERα/PR(A+B)/PRB expression in Normal women (n=58)

**Characteristcis**	**<or =50**	**>50**	***p* value**	***p* for Trend**
Number	50	8		
Age (n=58)	44.6±4.7	57.3±4.6	<0.001	
Age Group (n=58)			<0.001*	<0.001*
<50 y/o	42(84.0%)	1(12.5%)		
≧50 y/o	8(16.0%)	7(87.5%)		
Epithelium				
ERα expression, IRS (n=55)	0.5±0.8	0.1±0.4	0.176	
positive rate (%)	16/47 (34.0%)	1/8 (12.5%)	0.411	0.411
PR(A+B) expression, IRS (n=54)	0.3±0.6	0.1±0.4	0.464	
positive rate (%)	10/46 (21.7%)	1/8 (12.5%)	1.000	0.678
PRB expression, IRS (n=53)	0.4±0.9	0.5±0.9	0.878	
positive rate (%)	12/45 (26.7%)	2/8 (25.0%)	1.000	1.000
Stroma				
ERα expression, IRS (n=58)	1.0±1.1	0.6±0.9	0.420	
positive rate (%)	27/50 (54.0%)	3/8 (37.5%)	0.464	0.464
PR(A+B) expression, IRS (n=58)	1.5±1.4	0.5±0.8	0.055	
positive rate (%)	35/50 (70.0%)	3/8 (37.5%)	0.110	0.110
PRB expression, IRS (n=56)	1.7±1.3	1.0±0.8	0.174	
positive rate (%)	39/48 (81.3%)	6/8 (75.0%)	0.649	1.000

IRS: immunoreactive score = (intensity × percentage) of immunohistochemistry staining. Data are presented as number or mean ± standard deviation.

* p value < 0.05
