# Supplementary material for: Topological Distribution of the Sex Hormone Receptor Expressions Highlights the Importance of Stromal ERα and Epithelial PR in Malignant Transformation of the Uterine Cervix
Source: Int J Mol Sci. 2025 May 6;26(9):4418. doi: 10.3390/ijms26094418 (PMC12073007; doi:10.3390/ijms26094418)
Supplement: Supplementary file 1 [file ijms-26-04418-s001.zip › Table S2.pdf]

Table S2. Epithelium/carcinoma ERα/PR(A+B)/PRB expression in pre- and postmenopausal patients

	**Normal (n=58)**			**CIN2/3 (n=44)**			**CIS (n=70)**			**ICC (n=159)**		
	Menopausal status											
**Characteristic**	No	Yes	*p* value	No	Yes	*p* value	No	Yes	*p* value	No	Yes	*p* value
Number	50	8		23	21		35	35		54	105	
Age	44.6±4.7	57.3±4.6	<0.001*	40.7±3.2	54.1±5.1	<0.001*	39.1±6.2	63.9±10.4	<0.001*	43.2±6	66.9±12.3	<0.001*
Age Group												
<50 y/o	42(84.0%)	1(12.5%)		23(100.0%)	6(28..6%)		35(100.0%)	1(2.9%)		48(88.9%)	10(9.5%)	
≧50 y/o	8(16.0%)	7(87.5%)		0(0.0%)	15(71.4%)		0(0.0%)	34(97.1%)		6(11.1%)	95(90.5%)	
Epithelium												
ER α expression, IRS	0.5±0.8	0.1±0.4	0.176	1.0±1.4	0.4±0.8	0.158	1.0±1.2	1.2±1.6	0.606	0.3±0.6	1.0±2.1	0.018*
positive rate (%)	16/47 (34.0%)	1/8 (12.5%)	0.411	8/18 (44.4%)	3/15 (20.0%)	0.138	22/35 (62.9%)	21/35 (60.0%)	0.806	11/52 (21.2%)	31/100 (31.0%)	0.198
PR(A+B) expression, IRS	0.3±0.6	0.1±0.4	0.464	0.6±0.7	0.1±0.3	0.017*	0.1±0.4	0.1±0.4	0.554	0.2±0.9	0.0±0.2	0.069
positive rate (%)	10/46 (21.7%)	1/8 (12.5%)	1.000	9/21 (42.9%)	1/15 (6.7%)	0.024*	4/34 (11.8%)	2/34 (5.9%)	0.673	4/51 (7.8%)	2/96 (2.1%)	0.183
PRB expression, IRS	0.4±0.9	0.5±0.9	0.878	0.7±0.8	0.1±0.5	0.039*	0.2±0.7	0.2±0.5	0.635	0.1±0.4	0.1±0.4	0.786

positive rate (%)	12/45 (26.7%)	2/8 (25.0%)	1.000	9/20 (45.0%)	1/15 (6.7%)	0.022*	6/35 (17.1%)	5/35 (14.3%)	0.743	4/54 (7.4%)	8/100 (8.0%)	1.000

IRS: immunoreactive score = (Intensity × Percentage) of immunohistochemistry staining

Data are presented as number or mean ± standard deviation.

*Secretory phase vs. non-secretory phase
